# Targeting the DNA damage response for cancer therapy

**DOI:** 10.1042/BST20220681

**Published:** 2023-01-06

**Authors:** Nicola J. Curtin

**Affiliations:** Translational and Clinical Research Institute, Newcastle University Centre for Cancer, Faculty of Medical Sciences, Newcastle University, Newcastle upon Tyne NE2 4HH, U.K.

**Keywords:** ATM, ATR, CHK1, DNA damage response, DNA-PK, PARP

## Abstract

The DNA damage response (DDR) is an elegant system, coordinating DNA repair with cell cycle checkpoints, that evolved to protect living organisms from the otherwise fatal levels of DNA damage inflicted by endogenous and environmental sources. Since many agents used to treat cancer; radiotherapy and cytotoxic chemotherapy, work by damaging DNA the DDR represents a mechanism of resistance. The original rational for the development of drugs to inhibit the DDR was to overcome this mechanism of resistance but clinical studies using this approach have not led to improvements in the therapeutic index. A more exciting approach is to exploit cancer-specific defects in the DDR, that represent vulnerabilities in the tumour and an opportunity to selectively target the tumour. PARP inhibitors (PARPi) selectively kill homologous recombination repair defective (HRD, e.g. through *BRCA* mutation) cells. This approach has proven successful clinically and there are now six PARPi approved for cancer therapy. Drugs targeting other aspects of the DDR are under pre-clinical and clinical evaluation as monotherapy agents and in combination studies. For this promising approach to cancer therapy to be fully realised reliable biomarkers are needed to identify tumours with the exploitable defect for monotherapy applications. The possibility that some combinations may result in toxicity to normal tissues also needs to be considered. A brief overview of the DDR, the development of inhibitors targeting the DDR and the current clinical status of such drugs is described here.

## Introduction

Maintenance of DNA integrity is essential for the survival of all living organisms. The level of DNA damage inflicted from endogenous and environmental sources would be lethal without the DNA damage response (DDR). The DDR evolved as an elegantly orchestrated network of pathways that repair the DNA and arrest the cell cycle such that damage is neither copied during S-phase nor passed on to daughter cells during mitosis.

DDR dysfunction causes genomic instability and mutations that can ultimately lead to cancer formation [1]. Several components of the DDR are recognised as tumour suppressors, e.g. p53, BRCA1. In addition, many oncogenes, by forcing cells into S-phase before they are ready, cause replication stress (RS), which causes interruptions in the DNA sequence and abnormal DNA structures that require the DDR for their resolution (reviewed in [2]).

Dysfunction of one DDR pathway may lead to increased activity of compensatory pathways, resulting in resistance to DNA damaging chemotherapy and radiotherapy. The initial rationale for the development of drugs targeting the DDR was to overcome these resistance mechanisms. However, the strategy of inhibiting the DDR to chemo- or radiosensitise tumours also increases the toxicity to the normal tissues in line with increased antitumour activity such that there is only a minor impact, if any, on the therapeutic index. A much more exciting approach is to exploit the vulnerability created by the DDR defect to specifically target the tumour without harming normal tissues that have properly functioning DDR pathways (reviewed in [3]). This approach is based on the synthetic lethality concept where a defect in one of 2 complementary pathways does not affect viability but a defect in both is lethal. That this can work in the clinical setting is demonstrated by the remarkable success of PARP inhibitors (PARPi) in ovarian and other cancers associated with defects in homologous recombination DNA repair (HRR) (reviewed in [4]). This review describes the different types of DNA damage and the cellular response, the development of drugs targeting the DDR and the current clinical status of such drugs.

## Types of DNA damage, corresponding repair and cell cycle checkpoint signalling pathways

### DNA single strand damage

The most common types of DNA damage affect only one strand and are generally repaired rapidly so generally less critical to the survival of the cell than those lesions affecting both strands. Signalling to cell cycle checkpoints via p53 may occur [5,6]. The simplest type of repair is direct reversion of the damage eg, by methylguanine methyltransferase (MGMT), which removes methyl groups on guanine that were added accidentally by S-adenosylmethionine, environmentally by dietary nitrosamines or deliberately by anticancer DNA alkylating agents [7].

### Base damage and single strand breaks (SSBs)

**Figure 1.**
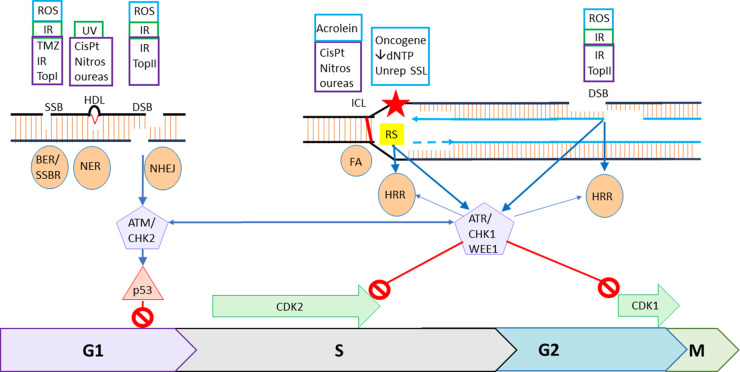
The DNA damage response: integration of repair and cell cycle checkpoints. Endogenous DNA damage is shown in blue boxes, environmental in green boxes and therapeutically induced in purple boxes. ROS (reactive oxygen species), IR (irradiation) TMZ (temozolomide) and other DNA methylating agents and TopI (topoisomerase I poisons) result in damaged bases and SSBs. BER/SSBR (base excision repair/single strand break repair) repair these lesions by removing damaged bases by glycosylase and endonuclease action to produce a SSB. PARP binds to and is activated by the nick to recruit the SSB repair proteins XRCC1, PolB and Ligase 1 or 3 to fill the gap and join the ends, for short patch repair with the additional requirement for PNPK, PCNA and FEN1 for long patch repair. HDL (Helix distorting lesions) caused by UV photoproducts, bulky adducts, e.g. from tobacco smoke and platinum therapy are repaired by NER (nucleotide excision repair) which can be global or transcription-coupled and involves several proteins including TFIIH XPG and XPF-ERCC1 to remove 25–30 nt, re-filling the gap using the complementary strand as a template by DNA pol δ, ε or κ and ligation by Lig 1 or 3. TopII (topoisomerase II poisons) along with ROS and IR cause DSBs that are repaired in GI exclusively by NHEJ (non-homologous end-joining) which involves the recognition, stabilisation and synapsis of the break by DNA-dependent protein kinase DNA-PK, a heterodimeric complex of KU70 and Ku80 and the catalytic subunit, DNA-PKcs, modest end resection, limited by 53BP1, and end ligation by Ligase 4 and XRCC4-XLF. DSBs signal via ATM and CHK2, primarily but not exclusively via p53, to G1 cell cycle arrest. ICL (interstrand cross-links) induced endogenously and environmentally by, e.g. acrolein and therapeutically by CisPt (*cis*- or carboplatin) are repaired by the FA (Fanconi anaemia) pathway when the replication fork encounters the lesion. The ICL is recognised by FANCM then recruitment of the core FA proteins for fork stabilisation, nucleases to excise the lesion then translesional and/or homologous recombination DNA synthesis. Several FA components are in common with NER (FANCM/ERCC1/XPF family) and HRR (homologous recombination repair), including BRCA2/FANCD1, PALB2/FANCN, RAD51C/FANCO. Oncogene activation and dNTP depletion and unrepaired (SSL) single strand lesions result in RS (replication stress) that can cause stalled/collapsed replication forks are repaired by HRR. Similarly DSBs are repaired by HRR during S phase. HRR involves end-resection involving MRN and CtIP, promoted by BRCA1, to produce single strand overhangs, BRCA2 recruits RAD51 to allow invasion of the complementary chromatid template for faithful DNA synthesis across the break. The process is completed by resolution of Holliday junctions by helicases and synthesis-dependent strand annealing. NER, FA, stalled replication forks and resected DSBs all result in SS DNA, which activate ATR, that activates CHK1, and WEE1 ultimately resulting in increased inactivating phosphorylation of the S and G2/M cyclin dependent kinases CDK2 and CDK1 causing S-phase and G2/M cell cycle arrest. There is cross-talk between ATR/CHK1/WEE1 and FA/HRR and ATM/CHK2 and NHEJ to ensure co-ordination between repair and cell cycle arrest.

Base modifications and nicks in the DNA backbone, usually resulting from oxidative stress are estimated to occur between 10 000 and 100 000 times per genome per day. This rate is increased by inflammation, which is a promoter of carcinogenesis [8]. These lesions are repaired by the base excision repair (BER)/single strand break repair (SSBR) pathway. BER encompasses the whole pathway, including removal of the damaged base and endonuclease digestion to create the SSB, and SSBR is just that downstream of the SSB. SSBs can also be directly induced by ROS or topoisomerase I. SSBR involves poly(ADP-ribose) polymerases, PARP1 or PARP2 to recognise the damage and recruit the repair proteins (Figure 1).

### Helix-distorting DNA lesions

Exposure to UV light is another common source of damage with estimates of as many as 100 000–200 000 DNA photoproducts generated by an hour's exposure to bright sunlight [9]. These are dealt with efficiently by the nucleotide excision repair (NER) pathway comprising ∼30 proteins, which also recognises bulky adducts on DNA, e.g. those caused environmentally by exposure to smoke and therapeutically with platinum agent chemotherapy. Global NER is the most common pathway but in active genes, transcription-coupled NER can be used. These pathways only differ in their lesion recognition step, thereafter the process is the same with the offending lesion and surrounding 25–30 nt being removed, the gap re-filled using the complementary strand as a template followed by ligation (Figure 1). Defects in XP genes, key components of NER, are associated with extreme photosensitivity and skin cancer [10].

### DNA mismatches

Errors in DNA, including a mis-matched base or, particularly in repetitive sequences (microsatellites), the skipping of bases or insertion of extra ones (Indels) may be introduced during replication. These are removed by the mis-match repair (MMR) pathway that includes MLH1 and MSH2 that are often mutated or silenced in cancers, particularly of the colon and endometrium. Defects in MMR leads to microsatellite instability and, because they act only on the daughter strand, cause tolerance to certain DNA lesions resulting in resistance to agents modifying the parental strand such as 6-thioguanine, DNA alkylating agents and platinums [11].

## Double strand damage

### DNA DSBs NHEJ and ATM signalling

DNA double strand breaks (DSBs), although rare, are much more catastrophic to the cell and more difficult to repair. DSBs can be caused by environmental ionising radiation, endogenous or environmental ROS, torsional strain during transcription or replication or if topoisomerase II is trapped on the DNA by some other lesion [12]. However, most DSBs occur during replication, e.g. through collision of a single-strand lesion with the replication fork or re-replication driven by oncogene activation [12] and will be discussed below.

DSBs occurring during the G1 phase of the cell cycle chiefly signal cell cycle arrest via the ATM–CHK2 (Ataxia telangiectasia mutated–Check 2 kinases) pathway and are largely repaired by non-homologous end joining (NHEJ) pathway (Figure 1) [3]. NHEJ is a rapid emergency response to DSBs and involves the recognition, stabilisation and synapsis of the break by DNA-dependent protein kinase DNA-PK, modest end resection, limited by 53BP1, may mean that some genetic material is lost and end ligation [13,14]. However, it is now thought that NHEJ is surprisingly accurate. DNA-PK phosphorylates repair components and itself to allow dissociation from the break and completion of repair. Loss of both ATM and DNA-PKcs is lethal [15]. ATM phosphorylates CHK2 and both kinases phosphorylate p53 to effect cell cycle arrest primarily at G1/S, ATM also phosphorylates H2AX and 53BP1 to promote repair of DSBs primarily via NHEJ [16]. ATM is considered as a tumour suppressor and patients with Ataxia telangiectasia (with homozygous *ATM* mutation) have extreme radiosensitivity, immunological deficiencies, neurodegeneration and cancer predisposition [17].

### DNA DSBs HRR and ATR–CHK1–WEE1 signalling

The most common endogenous source of DSBs is the collision of SSBs (and potentially other single strand lesions) with the replication fork. These lesions are generally repaired by the homologous recombination repair (HRR) also known as homology-directed repair (HDR) pathway. HRR is a complex pathway, using the sister chromatid as a template, resulting in high fidelity repair [13]. It is therefore restricted to S-phase and possibly G2. It involves end-resection promoted by BRCA1 thereby competing out 53BP1, which would otherwise restrict end resection and promote NHEJ that would be fatal with this type of DS end [13,18]. These resected DSBs also activate the DDR cell cycle checkpoint kinases: ATR, CHK1 and WEE1 [19]. BRCA2 recruits RAD51 to allow invasion of the complementary chromatid template for high fidelity template-directed repair (Figure 1). A back-up pathway to both NHEJ and HRR is alt-NHEJ, also known as microhomology-mediated end joining (MMEJ or polθ -mediated end joining (TMEJ)) [20].

The collision of unrepaired single-strand lesions with the replication fork along with replication stalling due to replication firing (often oncogene driven) with inadequate nucleotide supply causes RS [2]. RS is also resolved by HRR and is an important activator of ATR, CHK1 and WEE1 signalling to cell cycle arrest [21].

### Interstrand cross-links

Interstrand cross-links (ICLs) also rarely occur naturally, with endogenous sources being products of lipid peroxidation, e.g. acrolein and crotonaldehyde, which can also be generated in fatty food during cooking [22]. Repair of these lesions is by the Fanconi Anaemia (FA) pathway, which overlaps to a certain extent with both NER and homologous recombination repair (HRR). Repair of ICLs occurs when the replication fork encounters the ICL, involving FANCM recognition, recruitment of the core FA proteins, excision of the lesion and translesional and/or homologous recombination DNA synthesis (reviewed in [22]). FANCM also recruits ATR setting off the ATR-CHK1 cell cycle checkpoint signalling cascade [23].

### Targeting DNA repair

Since the DDR causes resistance to DNA damaging anticancer agents DDR inhibitors were first developed to overcome this resistance mechanism. MGMT inhibitors were developed to sensitise tumours to alkylating agent chemotherapy [24]. Pre-clinically these drugs were highly successful but disappointingly in the clinical setting they increased the toxicity of alkylating chemotherapy with minimal impact on antitumour activity and never progressed beyond Phase II trials [24].

Similarly PARP inhibitors (PARPi) were originally developed to prevent resistance to cytotoxic therapy-induced SSBs. Early inhibitors were developed using medicinal chemistry and an analogue by catalogue approach based around the by-product of the PARP reaction, nicotinamide [25,26]. They are well characterised pre-clinically for their ability to sensitise human cancer cells and tumours to DNA methylating agents, ionising radiation and topoisomerase I poisons [27]. In addition to preventing the resolution of SSBs, inhibited PARP1 is unable to dissociate from the DNA further obstructing DNA repair as first documented in 1992 [28]. Subsequently, it was reported that some PARPi were more effective trappers than others [29]), which is related to the potency of the inhibition [30]. The first clinical trial of a PARPi, rucaparib, was in combination with the DNA methylating agent temozolomide in 2003 [31]. Unfortunately, subsequent Phase II studies with this and other PARPi, e.g. olaparib, niraparib, veliparib in combination with temozolomide, topoisomerase I poisons and other cytotoxic drugs consistently resulted in increased toxicity and minimal antitumour benefit [32]. Even with tumour-directed radiotherapy the toxicity has been an issue [33].

The clinical experience with these early DDR inhibitors as chemo- and radiosensitisers has not lived up to the expectation from promising pre-clinical data. Neither MGMT nor PARP inhibitors have progressed beyond Phase II in combination with chemotherapy due to toxicity alongside only marginal improvements, if any, in efficacy.

A more promising approach is to target vulnerabilities specific to the tumour. Many DDR components are tumour suppressors and lost during carcinogenesis. In addition to creating vulnerability to DNA damaging anti-cancer therapies this can be exploited by targeting a complementary or compensatory DDR pathway to create synthetic lethality to kill the DDR-defective tumour cells. Since normal tissues have fully functional DDR pathways they will remain viable. This approach was first demonstrated in 2 landmark papers describing the synthetic lethality of PARPi in HRR defective (HRD, e.g. by *BRCA1* or *BRCA2* mutation) cells [34,35]. This is because unresolved SSBs, with or without trapped PARP1, accumulate and, during replication, result in aberrant DNA structures that can only be resolved by HRR [34]. *BRCA1* and *BRCA2* mutation carriers have a high risk of developing cancer, predominantly breast and ovarian but also prostate and pancreatic cancer [36]. Pre-clinical studies showed that the PARPi, rucaparib, was much more cytotoxic to cell lines and xenografts with *BRCA 1* or *BRCA2* mutations or epigenetically silenced *BRCA1* compared with wt cells or those with heterozygous *BRCA* mutations [37]. A key observation in this study was that a more continuous dosing schedule was more effective than a 5 or10-day dosing schedule

In 2009, the first of many PARPi monotherapy trials in patients with *BRCA* mutations confirming anticancer activity with minimal toxicity was published [38]. However, a study in patients with ovarian cancer revealed that *BRCA* mutations were not the perfect predictors of response to PARPi (olaparib in this case) [39]. Only ∼20% of ovarian cancer have germline or somatic *BRCA* mutations but an *ex-vivo* functional assay first demonstrated that ∼50–60% are HRD [40]. Commercial HRD tests (Myriad MyChoice CDX or Foundation Medicine's FoundationOne CDx) are frequently used as companion diagnostics, although they are not infallible [41,42]. The first FDA approvals of PARPi were in ovarian cancer with olaparib in 2014, followed by rucaparib and niraparib. Talazoparib was approved for breast cancer in 2018. PARPi are now also approved for castrate-resistant prostate cancer and pancreatic cancer. Two more PARPi have recently been approved for the treatment of ovarian cancer by China's NMPA: Fluzoparib and Pamiparib. The current clinical status of advanced PARPi is summarised in Table 1 and the subject of several reviews (e.g. [4,32,43,44]). Generally these inhibitors have little specificity for PARP1 over PARP2, which is highly similar structurally and functionally. However, recently a selective inhibitor of PARP1, AZD5305, more potent than olaparib pre-clinically and predicted to overcome some of the haematological toxicity associated with PARP2 inhibition, has entered clinical trials and the results are eagerly awaited [45].

**Table 1:** Current status (most advanced stage) of DDR inhibitors

Target	Drug	Current status	reference
PARP Approved and Phase III only	Olaparib/Lynparza®	**Approved (FDA)** as single agent since 2014 advanced BRCA mutated ovarian cancer^1^, BRCA mutated breast cancer (2018) pancreatic cancer (2019), BRCA or ATM mutated mCRPC^2^ (2020) gBRCA mutated HER2-ve high-risk early breast cancer (2022). **Approved** in combination with bevacizumab in ovarian cancer (2020) **Phase III** as single agent in ovarian cancer (e.g.NCT03534453, NCT03402841, NCT03106987,), Her2-ve & BRCA mutated breast cancer/TNBC^2^ (NCT03286842, NCT02032823). In combination with abiratrone or enzalutamide in mCRPC (NCT05171816, NCT03732820,, with cediranib or alpelisib or paclitaxel or durvalumab in ovarian cancer (NCT03278717, with pembrolizumab SCLC^2^ and NSCLC^2^, with bevacizumab in CRC^2^, with durvalumab in endometrial cancer	[92] [93]
	Rucaparib/Rubraca®	**Approved (FDA)** as single agent since 2016 in advanced BRCA mutated/HRD ovarian cancer BRCA mutated mCRPC (2020). **Phase III** as single agent in ovarian cancer, BRCA or ATM mutated mCRPC. In combination with nivolumab in ovarian cancer, with enzalutamide in mCRPC	[94] [95]
	Niraparib/Zejula®	**Approved (FDA)** as single agent since 2017 in advanced platinum-sensitive ovarian cancer **Phase III** as single agent in BRCAmut/HER2-ve mutated TNBC, In combination with Dostarlimab or bevacizumab or atezolizumab in ovarian cancer, with pembrolizumab in NSCLC, with abriratrone and prednisone in HRR gene mutated CRPC, in combination with Dostarlimab	[96] [97]
	Talazoparib/Talzenna®	**Approved (FDA)** as single agent since 2018 in BRCA mutated Her2-ve advanced breast cancer **Phase III** in combination with Avelumab in advanced ovarian cancer, and enzalutamide in mCRPC	[98] [99]
	Fluzoparib	**Approved (NMPA)** as single agent since 2020 in advanced platinum-sensitive ovarian cancer **Phase III** as single agent in platinum sensitive ovarian and pancreatic cancer, In combination with Apatinib in BRCA-mutated breast cancer	[100] [101]
	Pamiparib	**Approved (NMPA)** as single agent since 2021 in advanced BRCA-mutated ovarian cancer **Phase III** in combination with enzalutamide in mCRPC	[102] [103]
	Veliparib	**Phase III** in combination with carboplatin and paclitaxel in advanced ovarian cancer, and Her2-ve BRCA associated breast cancer	[104]
	IMP4297	**Phase III** as single agent in BRCA mutated ovarian cancer	[105]
DNA-PK	M3814/Peposertib/nedisertib	**Phase II** in combination with capecitabine and RT^2^ in CRC (NCT03770689) **Phase I** Single agent in solid tumours and CLL (NCT02316197) in combination with RT in locally advanced tumours (NCT02516813) gliomas (NCT04555577) H&N^2^ (NCT04533750), pancreatic cancer (NCT04172532), in combination with doxorubicin in ovarian cancer (NCT04092270), in combination with Avelumab and RT in solid tumours (NCT03724890), hepatobiliary cancer (NCT04068194) and mCRCP (NCT04071236) with mitoxantrone, cytarabine and etoposide in AML (NCT03983824)	[106]
	VX-984	**Phase I** alone and combination with doxorubicin (NCT02644278)	[107]
ATM	AZD0156	**Phase I** monotherapy or combination with olaparib, irinotecan or 5FU (NCT02588105)	[108]
	M4076	**Phase I** monotherapy (NCT04882917) in patients with solid tumours	[109]
	M3541	**Phase I** in combination with RT in patients with solid tumours (NCT03225105)	[110]
	AZD1390	**Phase I** monotherapy in patients with glioma (NCT05182905) in combination with radiotherapy in brain tumours (NCT03423628), lung cancer (NCT04550104) and sarcomas (NCT05116254,)	[111]
ATM/DNA-PK	XRD-0394	**Phase I** in combination with palliative RT in patients with locally advanced recurrent solid tumours (NCT05002140)	[112]
ATR	VE-822/VX-970/M6620/Bersosertib	**Phase II** monotherapy in Leiomyosarcoma and osteosarcoma (NCT03718091) as monotherapy or combination with gemcitabine in ovarian cancer (NCT02595892) or cisplatin and gemcitabine in urothelial cancer(NCT02567409) or NSCLC (NCT04216316) and in combination with topotecan in SCLC (NCT04768296), or with irinotecan in p53 mutated gastric cancer (NCT03641313), or sacituzumab govitecan in SCLC and PARP-resistant HRD tumours (NCT04826341) with carboplatin ± docetaxel in mCRPC (NCT03517969) and with lurbinectedin in SCLC (NCT04802174). Also in combination with Avelumab in tumours with various DDR defects (NCT04266912)	[113]
	AZD6738/ceralasertib	**Phase II** monotherapy or combination with olaparib in CCRC^2^, pancreatic and other cancers (***NCT03682289***^3^), In combination with olaparib in ovarian cancer (CAPRI: NCT03462342, ATARI: NCT04065269), SCLC (NCT03428607, NCT04699838, NCT02937818), BRCA mutated breast cancer (NCT04090567), TNBC (NCT03330847), mCRPC (TRAP: NCT03787680), osteosarcoma (NCT04417062) IDH-mutated tumours (NCT03878095). Also in combination with durvalumab in biliary tract (NCT04298008), SCLC (NCT04361825, NCT04699838) solid tumours and melanoma (NCT03780608, NCT05061134), with durvalumab and olaparib in TNBC (NCT03740893) NSCLC (NCT03334617) and in combination with durvalumab, olaparib or carboplatin in various advanced solid tumours (NCT02264678)	[114]
	BAY1895344 Elimusertib	**Phase II** monotherapy in pediatric patients with relapsed/refractory solid tumours (NCT05071209) **Phase I** monotherapy in advanced solid tumours and lymphomas (***NCT03188965***^3^). And combination with niraparib in ovarian cancer (NCT04267939), with gemcitabine in pancreatic, ovarian and other cancers (NCT04616534), with topoisomerase inhibitors (topotecan/irinotecan) in advanced solid tumours (NCT04514497) with FOLFIRI in advanced GI cancers (NCT04535401), with RT in H&N cancer (NCT04576091), with *cis*-or carboplatin and gemcitabine in urothelial cancer (NCT04491942) with pembrolizumab (NCT04095273) in advanced cancer	[115]
	VX-803/M4344	**Phase I** monotherapy (NCT02278250) or in combination with niraparib (NCT04655183- withdrawn in favour of M1774) in advanced solid tumours	[116]
	M1774	**Phase I** in combination with niraparib (***NCT04170153***^3^) and an immune checkpoint inhibitor (NCT05396833) in advanced solid tumours	[117]
CHK1	LY2606368/prexasertib	**Phase II** monotherapy in BRCA mutated ovarian and breast cancer and mCRPC (NCT02203513), in platinum-resistant ovarian cancer (NCT03414047) in tumours with RS or HRD (NCT02873975) in SCLC (NCT02735980) and in combination with irinotecan in DSRCT^2^ or RMS^2^ (NCT04095221)	[118]
	PF-477736	**Phase I** in combination with gemcitabine in advanced solid tumours (NCT00437203, terminated for business reasons)	[119]
	MK-8776/SCH900776	**Phase II** in combination with cytarabine in AML^2^ (NCT01870596) **Phase I** in combination with cytarabine in NCT00907517) or in combination with gemcitabine in leukaemia (NCT00779584)	[72] [73]
WEE1	AZD1775/MK-1775/Adavosertib	**Phase II** monotherapy in SCLC (NCT02593019, NCT02688907), BRCA mutated cancers (NCT04439227), uterine cancer (NCT04439227, NCT04590248), solid tumours with CCNE1 amplification (NCT03253679) or SETD2 mutation (NCT03284385). As monotherapy or in combination with cytarabine in AML (NCT03718143, NCT02666950), with gemcitabine in ovarian cancer (NCT02101775) and pancreatic cancer (NCT05212025), with gemcitabine and paclitaxel in pancreatic cancer (NCT02194829),and gemcitabine and RT in pancreatic cancer (NCT02037230), with carboplatin in ovarian cancer (NCT01164995) and other molecularly defined tumours (IMPACT: NCT01827384), in combination with cisplatin in TNBC (NCT03012477) and H&N cancer (NCT02087241), with carboplatin and paclitaxel in ovarian cancer ± p53 mutation (NCT01357161, NCT02272790), squamous cell lung cancer (NCT02513563) with docetaxel (NCT02087176) or pemetrexed and carboplatin (NCT02087241) in NSCLC, with paclitaxel in gastric adenocarcinoma with p53 mutation (NCT02448329), with olaparib in advanced solid tumours (NCT02576444) or ovarian cancer (NCT03579316, NCT03330847), with topotecan and cisplatin in cervical cancer (NCT01076400), with irinotecan in under 21 year olds (NCT02095132), Also with durvalumab and Tremelimumab, or carboplatin or olaparib in SCLC (NCT02937818)	[120]
	ZN-c3	**Phase I** monotherapy in advanced solid tumours including ovarian cancer (NCT05128825, NCT04158336, NCT04972422), TNBC and advanced ovarian cancer (NCT05368506), uterine serous carcinoma (NCT04814108). In combination with niraparib (NCT05198804), or carboplatin or paclitaxel or doxorubicin or gemcitabine (NCT04516447) in platinum-resistant ovarian cancer with gemcitabine in osteosarcoma (NCT04833582) and with bevacizumab ± pembrolizumab in metastatic CCNE1 amplified and TP53 mutant solid tumors (NCT05431582)	[121]
	IMP7068	**Phase I** monotherapy in advanced solid tumours (NCT04768868)	[122]
	SY-4835	**Phase I** monotherapy in advanced solid tumours (NCT05291182)	[123]
Polθ	ART4215	**Phase I** monotherapy and in combination with the PARPi, talazoparib or niraparib, in patients with advanced cancer. (NCT04991480)	[124]

1 Ovarian cancer refers to ovarian, fallopian and primary peritoneal cancer;

2 mCRPC, metastatic castrate-resistant prostate cancer; CRC, colorectal cancer; TNBC, triple negative breast cancer; SCLC, small cell lung cancer; NSCLC, non-small cell lung cancer; CCRC, clear cell renal cell cancer; DSRCT, Desmoplastic Small Round Cell Tumour; RMS, Rhabdomyosarcoma; AML, Acute myeloid leukaemia; RT, radiotherapy;

3 Study using ATM defect/deficiency as a biomarker for patient selection.

Anticancer radiotherapy and topoisomerase II poisons, such as doxorubicin, induce DSBs so NHEJ may cause resistance. DNA-PKcs, ATM, and ATR are members of the PI3-Kinase-like kinase (PIKK) family [46] and early DNA-PK inhibitors, developed from PI3K inhibitors, generally also inhibited the other PIKKs. The PI3K inhibitor, LY294002, was used as a lead for the development of more specific DNA-PK inhibitors: NU7026 and NU7441, and subsequently KU0060648 with *in vivo* activity [44]. Multiple pre-clinical studies demonstrate excellent radiosensitisation and sensitisation of topoisomerase II poisons, e.g,. etoposide and doxorubicin (reviewed in [47]). Pre-clinical data suggest resistance may arise via increased ATM signalling [48]. The first DNA-PK inhibitor to enter clinical trial was CC-115, a dual DNA-PK — mTOR inhibitor in 2011 (NCT01353625) [49]. More recently, clinical studies with more selective DNA-PK inhibitors, e.g. VX-984 and M3814 (peposertib, nedisertib, MSC2490484A) have been initiated (Table 1). Data on the Phase1 trial of peposertib (M3814, NCT02316197) identified that DNA-PK phosphorylation was reduced and 12 out of 31 patients had disease stabilisation for ≥12 weeks [50].

### Targeting cell cycle checkpoint signalling

ATM inhibitors have also been developed pre-clinically and progressed to clinical trial (reviewed in [51]). The first, KU55933, enhanced the sensitivity of cells to ionising radiation, topoisomerase I and II poisons [52]. Despite p53 being a major target of ATM, neither radiosensitisation nor chemosensitisation by KU55933 nor the more potent, KU59403, was p53 dependent [53]. Recent studies demonstrate that the novel ATM inhibitor AZD0156 also enhances the cytotoxicity of the PARPi, olaparib [54]. Several clinical trials of ATM inhibitors are underway (Table 1) alone or in combination with chemotherapy, radiotherapy, PARPi or immune checkpoint inhibitors and we await publication of the data.

Few CHK2 inhibitors have been developed; studies with early inhibitors suggests that they are radioprotective, consistent with the finding that *CHK2* knockout mice are radioresistant (reviewed in [19]). Clinical studies with the dual CHK1/CHK2 inhibitor AZD7762 were terminated due to cardiotoxicity [55].

The loss of G1 control, a common feature in cancer [56,57], and increased RS in tumours both result in dependency on ATR, CHK1 and WEE1 signalling, making these kinases attractive targets for cancer therapy. Studies with kinase dead ATR and caffeine (an early inhibitor) provided additional justification [58–60]. The first small molecule ATR inhibitor, NU6027, enhanced the sensitivity of several DNA damaging anticancer agents. NU6027 also inhibited HRR and sensitised cells to PARPi [61]. Other early ATR inhibitors AZ20 and AZD6738 sensitised cells to radiation, cisplatin and gemcitabine (reviewed in [19]) as did the potent and selective ATR inhibitor VE-821 [62,63]. VE-822 (VX-970, M6620, berzosertib) was the first ATR inhibitor to enter clinical trial, alone and in combination with radiotherapy, chemotherapy drugs and PARPi (Table 1). Berzosertib, AZD6738, M1774 and M4344 are all in clinical trial alone or in combination with gemcitabine, carboplatin or PARPi (Table 1) with some evidence of efficacy as summarised in [19]. In Phase II Berzosertib added benefit in combination with gemcitabine in platinum-resistant ovarian cancer [64] but not in advanced urothelial cancer [65]. AZD6738 showed some benefit in combination with radiotherapy [66] and the PARPi, olaparib [67] in PARPi-resistant ovarian cancer [68], and may improve anti-PD-1 therapy in melanoma and advanced gastric cancer [69,70]. M1774 and M4344 (VX-803) are also under investigation with the PARPi, niraparib, (Table 1) but no results are available yet.

The first potent and selective CHK1 inhibitor was PF-477736, others include MK-8776 (SCH900776), SRA737 and LY2606368 (prexasertib). In pre-clinical studies CHK1 inhibitors sensitise cells to radiation, various cytotoxic drugs and PARPi (reviewed in [19]). PF-477736, like the ATR inhibitor NU6027, also inhibited HRR suggesting that this was responsible for the synergy with PARPi [71]. Both MK8776 and prexasertib (LY2606368) are under clinical investigation (Table 1). MK8776 showed some evidence of activity in Phase I [72] but no improvement of cytarabine response in Phase II [73]. Prexasertib is in multiple clincal trials as monotherapy, particularly in HRD tumours or those with RS (Table 1). Early studies suggest it is tolerable with activity in platinum-resistant *BRCA* wt ovarian cancer and triple negative breast cancer [74,75].

AZD1775/MK-1775 is the only WEE1 inhibitor in clinical trial where there is published pre-clinical data. Pre-clinically it increases the activity of multiple DNA damaging agents (reviewed in [19]) There are several ongoing trials of this and other WEE1 inhibitors (Table 1). However, only a Phase 0 trial to demonstrate brain penetration of AZD1775 has been published [76].

The success of the synthetic lethality with PARPi in HRD cancer stimulated the search for determinants of sensitivity to other DDR inhibitors that could be used as predictive biomarkers. Screens with knock-down/deletion of various genes offer great opportunities to identify predictive biomarkers, although often the complicated molecular pathology of cancer means they have not lived up to expectation [77,78]. In addition, different surrogate methods of measuring cell viability can give different estimates of cytotoxicity [79]. Nevertheless defects/deficiency in ATM seem likely to be predictive of response to ATR inhibitors and are being investigated in several studies (Table 1). RS also has the potential to predict sensitivity to ATR, CHK1 and WEE1 inhibitors. Since RS can be estimated from γH2AX foci, including in FFPE material, it may be a useful biomarker (reviewd in [80])

### Targeting resistance to DDR inhibition

Development of resistance to PARPi is common, usually through the restoration of HRR function, e.g. by secondary mutation in the *BRCA* gene that restores its function [81] or the loss of *53BP1* (which competes with BRCA1 to promote what would be fatal NHEJ over HRR) [4,82,83]. Ways to overcome this are being sought. ATR or CHK1 inhibition, whether by abrogation of cell cycle arrest or inhibition of HRR [61,84–86] is a promising approach. Several clinical trials of PARP and ATR inhibitors have completed or are ongoing including the ATARI, CAPRI and TRAP trials of AZD6738 with olaparib (Table 1) and M6620 and veliparib in combination with cisplatin [87]. The CHK1 inhibitor, prexasertib in combination with olaparib has also been evaluated (Table 1) and the findings are eagerly awaited.

Inhibiting polθ to target MMEJ, the back-up pathway for HRR and NHEJ, may be another way to overcome PARPi resistance. Polθ has both ATPase and polymerase function and is an emerging target in cancer therapy [20] including for radiosensitisation [88]. Polθ inhibitors targeting both the ATPase and polymerase functions are under development [89]. The first to enter clinical trial is the polymerase inhibitor ART4215 in 2021 (Table 1). No pre-clinical data are available with these inhibitors but novobiocin, which also inhibits polθ, is synthetically lethal in HRD tumours [90] and with DNA-PK inhibition [91].

## Perspectives

- The DDR is essential for life, with overlapping and complementary well-coordinated pathways of repair and cell cycle checkpoints. These pathways, evolved to enable survival from endogenous and environmental DNA damage, confer resistance to DNA damaging anticancer agents. DDR defects are also common in cancer. Drugs targeting the DDR have significant potential to overcome resistance and exploit DDR defects. The discovery of the synthetic lethality of PARPi in HRD cancers, first published in 2005, was validated clinically 4 years later and now 6 PARPi are approved using this approach.
- The approach of sensitising cancers to DNA damaging therapy with DNA repair inhibitors, although hugely promising in pre-clinical studies, has been disappointing clinically. The regulatory approval of PARPi monotherapy in patients with HRD tumours has massively stimulated research in DDR inhibitors, particularly those targeting ATR and CHK1, which also have the potential to reverse PARPi resistance.
- Exploiting cancer-specific DDR defects by targeting other aspects of the DDR has immense potential for tumour-selective therapy with minimal toxicity. Reliable, robust biomarkers to identify tumour vulnerabilities are needed. Identification of determinants of sensitivity/predictive biomarkers using genetically modified cells may not translate to the complex phenotype of cancer cells. It is tempting to speculate that, since most tumours have dysregulated G1 cell cycle control, which may be exploited by targeting intra S and G2M checkpoints to selectively target a tumour vulnerability, ATR, CHK1 and WEE1 inhibitors may prove the most successful either as single agents or in combination. As Polθ is up-regulated in many tumours, and MMEJ is a back-up for both HRR and NHEJ, this is a potentially promising target.The genomic instability of tumour cells means that resistance is likely to develop. Combining different DDR inhibitors may reduce this but there are potential risks that the tumour-specificity will be lost and the combination will be toxic. Combinations should be guided by the evidence from careful and extensive *in vivo* pre-clinical investigation of proposed combinations prior to experimenting on patients.

## Abbreviations

BER: base excision repair
DDR: DNA damage response
DSBs: double strand breaks
FA: Fanconi Anaemia
HRR: homologous recombination DNA repair
ICL: interstrand cross-links
MGMT: methylguanine methyltransferase
MMEJ: microhomology-mediated end joining
MMR: mis-match repair
NER: nucleotide excision repair
NHEJ: non-homologous end joining
PARPi: PARP inhibitors
RS: replication stress
SSBR: single strand break repair
